# Epigenetic Regulation Associated With Sirtuin 1 in Complications of Diabetes Mellitus

**DOI:** 10.3389/fendo.2020.598012

**Published:** 2021-01-18

**Authors:** Jie Wang(a), Shudong Wang, Jie Wang(b), Mengjie Xiao, Yuanfang Guo, Yufeng Tang, Jingjing Zhang, Junlian Gu

**Affiliations:** ^1^ School of Nursing, Cheeloo College of Medicine, Shandong University, Jinan, China; ^2^ Department of Cardiology at the First Hospital of Jilin University, Changchun, China; ^3^ Department of Orthopedic Surgery, The First Affiliated Hospital of Shandong First Medical University, Jinan, China; ^4^ Department of Cardiology at the First Hospital of China Medical University, and Department of Cardiology at the People’s Hospital of Liaoning Province, Shenyang, China

**Keywords:** diabetes mellitus, diabetic complications, SIRT1, epigenetics, deacetylation

## Abstract

Diabetes mellitus (DM) has been one of the largest health concerns of the 21st century due to the serious complications associated with the disease. Therefore, it is essential to investigate the pathogenesis of DM and develop novel strategies to reduce the burden of diabetic complications. Sirtuin 1 (SIRT1), a nicotinamide adenosine dinucleotide (NAD^+^)-dependent deacetylase, has been reported to not only deacetylate histones to modulate chromatin function but also deacetylate numerous transcription factors to regulate the expression of target genes, both positively and negatively. SIRT1 also plays a crucial role in regulating histone and DNA methylation through the recruitment of other nuclear enzymes to the chromatin. Furthermore, SIRT1 has been verified as a direct target of many microRNAs (miRNAs). Recently, numerous studies have explored the key roles of SIRT1 and other related epigenetic mechanisms in diabetic complications. Thus, this review aims to present a summary of the rapidly growing field of epigenetic regulatory mechanisms, as well as the epigenetic influence of SIRT1 on the development and progression of diabetic complications, including cardiomyopathy, nephropathy, and retinopathy.

## Introduction

Diabetes mellitus (DM) refers to abnormalities in the metabolic processing of carbohydrates, fats and proteins that is characterized by persistent hyperglycemia as a result of insufficient insulin secretion, impaired insulin action or both (1). DM has gradually become one of the largest health concerns of the twenty-first century, and affects approximately 451 million adults worldwide, with projections that the disease will reach 693 million adults by 2045 (2). The majority of DM-related rates of morbidity and mortality are due to serious complications resulting from chronic hyperglycemia, including cardiovascular disorders, nephropathy, retinopathy with potential loss of vision, peripheral neuropathy and autonomic neuropathy (3). These long-term complications make DM a considerable burden to public health since they require lifelong care and treatment. Therefore, it is essential to investigate the disease pathogenesis and develop novel therapeutic strategies to reduce the burden of DM and its associated complications.

DM is a hereditary disease with a strong genetic predisposition (4). Interestingly, in addition to genetics, environmental factors including less exposure to sunlight and lack of physical activity also increase the risk of DM (5). However, numerous people exposed to these risks do not go on to develop DM. Indeed, strong evidence has shown that epigenetics plays a significant role in the complex interaction between genes and the environment (6). Epigenetics is described as stable and heritable alterations in gene expression or cellular phenotypes without changes in nucleotide sequences, and includes DNA methylation, histone modifications and non-coding RNA (ncRNA)-mediated pathways (7). Recently, a growing number of studies have focused on the role of epigenetics in DM and its related complications.

Sirtuin 1 (SIRT1), a nicotinamide adenosine dinucleotide (NAD^+^)-dependent deacetylase, has been reported to deacetylate histones to modulate chromatin function, such as histone 3 lysine 9 (H3K9) and H4K16 (8). SIRT1 can also deacetylate numerous transcription factors to regulate the expression of target genes either positively or negatively, including tumor protein 53 (p53) (9), forkhead box O (FOXO) (10), nuclear factor kappa B (NF-κB) (11), and peroxisome proliferator-activated receptor gamma coactivator-1 alpha (PGC-1α) (12). Moreover, SIRT1 plays a crucial role in regulating histone and DNA methylation through the recruitment of other nuclear enzymes to the chromatin (8, 13). All above mentioned findings suggest that SIRT1 is a crucial regulator of epigenetics. Furthermore, SIRT1 acts as a cellular energy sensor and plays a pivotal role in regulating energy metabolism, insulin sensitivity, and cardiovascular functions in mammals (14–16). Recently, numerous studies have concentrated on the key roles of SIRT1 and related epigenetic mechanisms in diabetic complications. Therefore, the primary purpose of this review is to summarize the recent advances regarding the effects and related epigenetic mechanisms of SIRT1 in diabetic complications, including diabetic cardiomyopathy (DCM), diabetic nephropathy (DN), and diabetic retinopathy (DR). Finally, we will list the SIRT1 agonists that have been reported to exert protective functions during the development of DM-associated complications.

## Epigenetic Mechanisms That Influence Diabetic Cardiomyopathy

DCM is one of the major diabetic complications characterized by structural abnormalities and ventricular systolic and/or diastolic dysfunction in the absence of hypertension, coronary artery disease or significant congenital cardiac diseases (17). Accumulating evidence has revealed an association between “hyperglycemic memory” and epigenetic mechanisms (18), which may exert significant roles in the development of DCM. Hyperglycemic memory, also known as metabolic memory, is a phenomenon where the negative effects of hyperglycemia persist even after glycemic control has been achieved. Yu et al. explored the effect of H3K9 trimethylation (H3K9me3), a key epigenetic chromatin marker, on high glucose (HG)-induced inflammation and metabolic memory in rat cardiomyocyte cell line H9c2. They found that HG conditions increased the levels of the inflammatory cytokine interleukin-6 (IL-6) and decreased the levels of H3K9me3 at the *IL-6* promoter, which could not be reversed following the removal of HG stimulation. These results indicated that decreased levels of H3K9me3 at the *IL-6* promoter following HG exposure was a main mechanism that affected hyperglycemic memory in cardiomyocytes (19).

Moreover, histone deacetylase (HDAC)-mediated epigenetic processes also have important functions in the modulation of DCM. Chen et al. investigated the role of HDAC in DCM and found HDAC inhibition could attenuate cardiac hypertrophy, interstitial fibrosis and apoptosis accompanied by increased acetylation of glucose transporter 1 (GLUT1) and phosphorylation of p38 in diabetic mice (20). p38 phosphorylation has been verified to be involved in cardioprotection induced by glucagon-like peptide-1 in myocardial ischemia and reperfusion injury (21). However, whether GLUT1 acetylation mediates its physiological functions needs to be further explored. Peroxisome proliferator-activated receptors (PPARs) have emerged as critical regulators of cardiac glucose and lipid homeostasis. HDAC inhibition in a rat model of DM increased the expression of cardiac PPAR-α and PPAR-δ but decreased cardiac PPAR-γ expression compared with untreated DM rats, suggesting that HDAC inhibition could regulate fatty acid metabolism to improve DCM (22). Previous studies have only focused on the total inhibition of HDAC activity using a non-specific HDAC inhibitor. Recently, Xu et al. explored the effect of specific HDAC inhibitor on DCM and found that HDAC3 inhibition by RGFP966 could protect against DM-induced cardiac remodeling and dysfunction in diabetic mice. Furthermore, RGFP966 could repress extracellular signal-regulated kinases 1/2 (ERK1/2), a well-known initiator of cardiac hypertrophy. This effect was mediated by increased dual specificity phosphatase 5 (DUSP5) expression through the acetylation of histone H3 on the primer region of the *DUSP5* gene, a nuclear phosphatase of ERK1/2 (23). Contrary to the above, silent information regulator 2 (Sir2), an NAD^+^-dependent HDAC, has been reported to have a beneficial effect on DCM. A study by Dong et al. demonstrated that fidarestat, an aldose reductase inhibitor, could ameliorate cardiomyocyte dysfunction in diabetic obese mouse cardiomyocytes through regulation of Sir2 expression (24).

Emerging evidence has shown that microRNAs (miRNAs) are implicated in the maintenance of tissue homeostasis in the heart. A study on the alteration of miRNAs expression in a mouse model of DM revealed that 316 out of 1,008 total miRNAs were disordered in the diabetic heart (25). Of note, microRNA-212 (miR-212) and miR-221 were massively overexpressed in the diabetic heart, which could play pivotal roles in cardiac hypertrophy and autophagy, and glycemic control was unable to restore the levels of these two miRNAs to normal (25–27). Similarly, the expression of miR-199a has been reported to be upregulated in association with myocardial hypertrophy, whereas miR-30a and miR-1 (potent inhibitors of mitochondrial fission, hypertrophy and apoptosis), as well as miR-29b (anti-fibrosis) were downregulated in diabetic mouse heart (25). Moreover, the expression of miR-451, miR-144, and miR-133a was also shown to be altered in diabetic murine hearts, and involved in the development of DCM (28–30). Exploring the epigenetic mechanisms of miR-133a found that it could regulate DNA methylation in diabetic mouse cardiomyocytes (30).

Long ncRNAs (lncRNAs), another type of ncRNA, have also been considered as a novel therapeutic strategy for the treatment of DCM. Silencing lncRNA Kcnq1ot1 decreased caspase-1 expression and repressed transforming growth factor-beta 1 (TGF-β1) signaling to improve pyroptosis and fibrosis in a mouse model of DCM (31). Moreover, downregulation of lncRNA metastasis-associated lung adenocarcinoma transcript 1 (MALAT1) and myocardial infarction associated transcript (MIAT) improved DCM by reducing apoptosis and ameliorating cardiac function (32, 33). In contrast, downregulation of lncRNA H19 by small interfering RNA (siRNA) decreased miR-675 expression and in turn upregulated the expression of voltage-dependent anion channel 1, which consequently promoted cellular apoptosis in neonatal rat cardiomyocytes exposed to HG conditions (34). The altered expression levels of the ncRNAs discussed above might explain why DM-related myocardial injury still progressively worsens even in the presence of glycemic control.

In addition to histone post-translational modifications and ncRNA-mediated pathways, the effects of DNA methylation on the development of DCM have also been reported. Mönkemann et al. investigated the epigenetic alteration of *p21^WAF1/CIP1^* and cyclin D_1_ gene expression involved in cell cycle control in cardiomyocytes of streptozocin (STZ)-induced diabetic rats. They found that the p53-inducible *p21^WAF1/CIP1^* gene was completely demethylated and activated, while the cyclin D_1_ gene was completely methylated and inactivated. These different methylation patterns might be the result of p53-dependent cell cycle arrest associated with DNA damage. Activated p21^WAF1/CIP1^ suppresses DNA replication by binding proliferating cell nuclear antigen, which is necessary to both replicative DNA synthesis and DNA repair (35). As discussed above, epigenetic changes are closely related to the development of DCM, and a complete understanding of the roles of epigenetic processes in DCM would provide novel insights into this complex disease.

## SIRT1 in Diabetic Cardiomyopathy

Several studies have shown the possibility that SIRT1, as a class-III HDAC, exerts a protective effect on DCM by deacetylating histones. NF-κB, a pleiotropic transcription factor, can be translocated to nucleus to induce transcription of several proinflammatory genes under oxidative stress. In diabetic rats, SIRT1 activation by resveratrol could reduce the acetylation of the p65 subunit of NF-κB (NF-κB-p65) at K310. NF-κB-dependent transcription is associated with acetylation status of histone. Indeed, increased acetylation of H3K9 was observed in the hearts of diabetic rats, which was reduced after resveratrol administration (11). Based on the above findings, we propose that increased SIRT1 expression may be responsible for the deacetylation of NF-κB-p65 and H3K9, as well as the reduced NF-κB-p65-mediated actions in the diabetic heart. In addition, p66Shc expression was upregulated in the diabetic heart, which was related to oxidative stress, myocardial inflammation, and cardiac dysfunction. Importantly, three-week intensive glycemic control was unable to revert the above-mentioned phenomenon. This study focused on the epigenetic regulation of the prooxidant adaptor p66Shc in the hearts of diabetic mice and found that dysregulation of DNA methyltransferase 3b (Dnmt3b) and deacetylase SIRT1 resulted in CpG demethylation and histone H3 acetylation at the *p66Shc* promoter, which could lead to sustained transcription of the *p66Shc* (36).

Other than histone deacetylation, it has been widely reported that SIRT1 is able to deacetylate a wide range of non-histone substrates to result in activation or repression of their catalytic activity in DCM. PGC-1α, as a master regulator of mitochondrial biogenesis, can be acetylated by HG treatment. However, resveratrol decreased the acetylation of PGC-1α in HG-treated H9c2 cells. Additional studies investigating the underlying mechanisms found that the resveratrol-induced PGC-1α deacetylation depended on SIRT1. Meanwhile, resveratrol improved mitochondrial function to alleviate DCM by increasing SIRT1 expression and PGC-1α deacetylation (12). Another study by Ding et al. revealed that PGC-1α can directly bind to the dynamin-related protein 1 (*Drp1*) promoter to modulate protein expression. Melatonin reduces Drp1-mediated mitochondrial fission to exert its cardioprotective role in DCM through the SIRT1/PGC-1α signaling pathway (37).

Activation of poly (ADP-ribose) polymerase 1 (PARP1) is dependent on NAD^+^ and plays an important role in DNA repair and chromatin remodeling. In addition, PARP1 is also involved in transcriptional regulation, telomere cohesion, and mitotic spindle formation during cell division, intracellular transport and energy metabolism (38). In a mouse model of type 2 diabetes (T2D), the mice exhibited increased oxidative stress, inflammation, cardiac hypertrophy, and fibrosis that was associated with enhanced PARP1 activity and decreased SIRT1 expression, while PARP1 inhibition could increase the levels of SIRT1 and PGC-1α to improve the above-mentioned adverse effects (39). 1,25-Dihydroxyvitamin D3 (1,25(OH)_2_D_3_) has been reported to be a potential PARP1 inhibitor. Treatment with 1,25(OH)_2_D_3_ can inhibit PARP1 expression to increase the expression of SIRT1 and repress the phosphorylation of mammalian target of rapamycin (mTOR), which improves cardiac dysfunction, hypertrophy and fibrosis in rats of type 1 diabetes (T1D) (40).

Autophagic dysfunction is a common occurrence in DM. Wang et al. investigated the mechanisms underlying the protective effect of resveratrol against heart failure and explored the role of SIRT1 in the modulation of autophagic flux in diabetic mice. They found that SIRT1 activation by resveratrol could deacetylate FOXO1 and enhance FOXO1 DNA binding at the Ras-related protein Rab-7 (*Rab7*, a crucial factor in the maturation of autophagosomes and their fusion with lysosomes) promoter region to ameliorate dysfunctional autophagic flux in the hearts of diabetic mice, suggesting that the effect of the SIRT1/FOXO1/Rab7 axis on autophagic flux may be a therapeutic strategy for the treatment of DCM (10).

It is commonly believed that histone deacetylation is tightly linked to DNA methylation. For example, the methyl-CpG-binding protein MeCP2 interacts specifically with methylated DNA and mediates transcriptional repression. Additionally, it was discovered that this repression by MeCP2 required a HDAC complex (41). Peng et al. reported that SIRT1 could deacetylate Dnmt1 to change its activity *in vitro* and *in vivo*. Specifically, deacetylation of Lys1349 and Lys1415 in the catalytic domain of Dnmt1 increases its methyltransferase activity. However, deacetylation of lysine residues in repeating glycine-lysine dipeptides (the GK linker) decreases Dnmt1’s methyltransferase-independent transcriptional repression (42). Recently, a study regarding the gestational DM-induced fetal programming of a heart ischemia-sensitive phenotype later in life found that cardiac oxidative stress and DNA hypermethylation in the offspring induced by gestational DM leaded to decreased expression of SIRT1 and aberrant development of the heart ischemia-sensitive phenotype (43). Therefore, it is easy to speculate that the crosstalk between the deacetylation of SIRT1 and DNA methylation may have an important role in DCM, and the mechanisms need to be further investigated.

**Figure 1:**
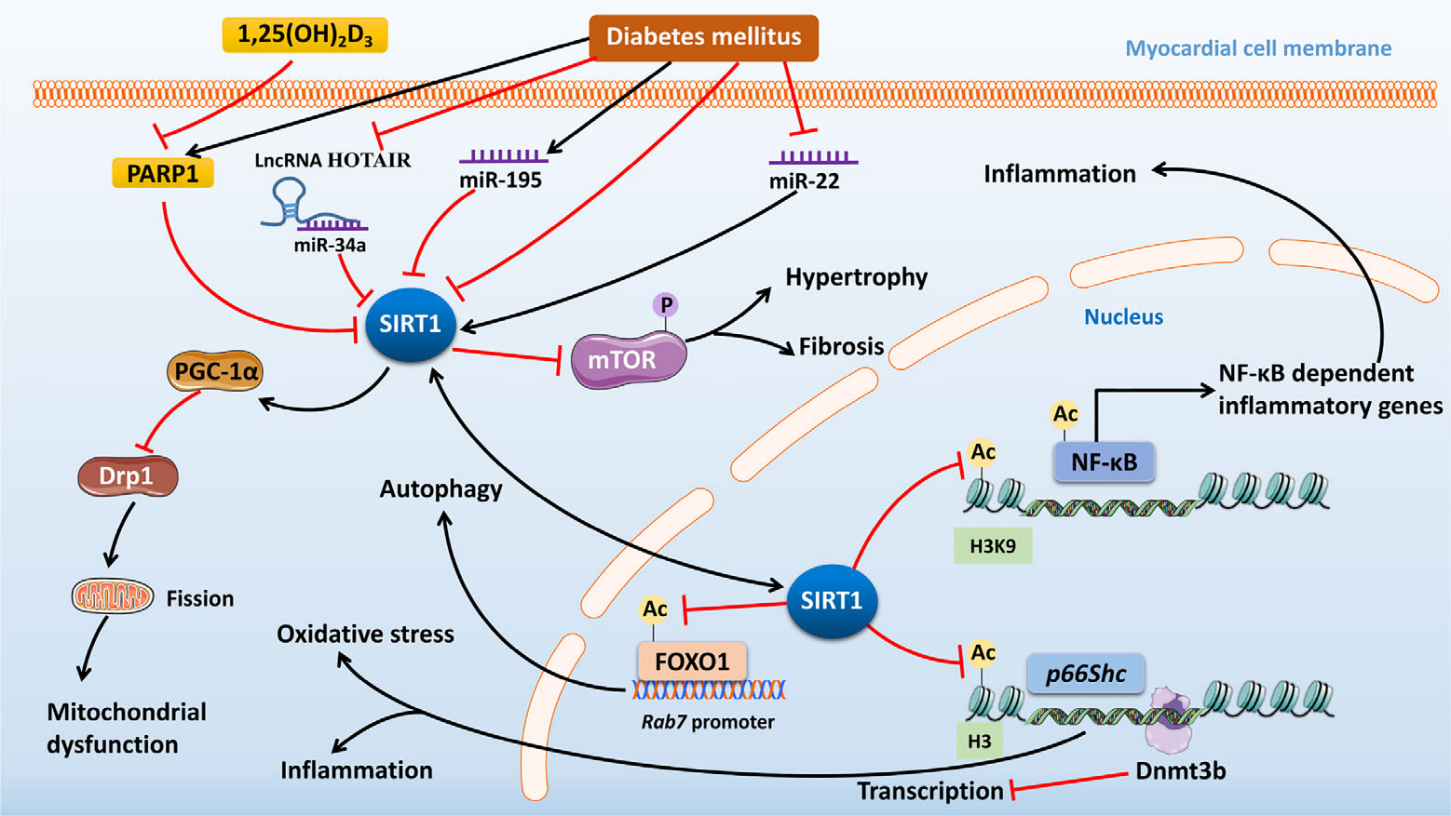
The mechanisms of action and signaling pathways of SIRT1 in diabetic cardiomyopathy. Diabetes mellitus (DM) can inhibit SIRT1 expression by increased expression of PARP1, miR-34a, and miR-195, or by decreased expression of miR-22. SIRT1 can deacetylate NF-κB and H3K9 to inhibit the expression of NF-κB-dependent inflammatory genes, which improves DCM. Dysregulation of Dnmt3b and SIRT1 by DM leads to CpG demethylation and histone H3 acetylation at the *p66Shc* promoter, which can result in sustained transcription of *p66Shc* to cause cardiac oxidative stress and inflammation. SIRT1 can reduce Drp1-mediated mitochondrial fission to exert its cardioprotective role in DCM through increasing PGC-1α expression. 1,25(OH)_2_D_3_ treatment can inhibit PARP1 expression to increase the expression of SIRT1 and repress the phosphorylation of mTOR, thus improving DM-induced cardiac hypertrophy and fibrosis. And SIRT1 can deacetylate FOXO1 and enhance FOXO1 DNA binding at the *Rab7* promoter region to ameliorate dysfunctional autophagic flux in the hearts of diabetic mice. Finally, lncRNA HOTAIR can upregulate SIRT1 expression by sponging miR-34a to improve DCM. SIRT1, Sirtuin 1; DM, diabetes mellitus; PARP1, poly (ADP-ribose) polymerase 1; miR, microRNA; NF-κB, nuclear factor kappa B; Dnmt3b, DNA methyltransferase 3b; DCM, diabetic cardiomyopathy; Drp1, dynamin-related protein 1; PGC-1α, peroxisome proliferator-activated receptor gamma coactivator-1 alpha 1,25(OH)_2_D_3,_ 1,25-Dihydroxyvitamin D3; mTOR, mammalian target of rapamycin; FOXO1, forkhead box O 1; lncRNA HOTAIR, long non-coding RNA HOX transcript antisense RNA.

Furthermore, SIRT1 is a direct target of many miRNAs. Suppression of miR-34a has been shown to restore the expression of SIRT1 to reverse the adverse epigenetic characteristics at *p66Shc* promoter in human cardiomyocytes exposed to HG conditions (36). Zheng et al. showed that the expression of miR-195 was upregulated while SIRT1 was downregulated in STZ-induced diabetic mouse heart. However, silencing of miR-195 expression inhibited oxidative stress, alleviated myocardial hypertrophy and improved cardiac function in diabetic mice, while at the same time the cardiac levels of B-cell lymphoma 2 (Bcl-2) and SIRT1 were upregulated (44). Therefore, it is possible that the beneficial effect of reduced miR-195 expression on DCM may be due to the upregulated expression of SIRT1. However, various miRNAs will have different effects on SIRT1, and overexpression of miR-22 upregulates SIRT1 to attenuate oxidative stress injury in a mouse model of DCM (45). LncRNA HOX transcript antisense RNA (HOTAIR), as a competing endogenous RNA, upregulates SIRT1 by sponging miR‐34a to improve DCM (46). Altogether, SIRT1 as a deacetylase plays a necessary role in the regulation of the epigenetics in DCM (**Figure 1**), and in-depth research will provide more information regarding potential therapeutic strategies to improve DCM in the future.

## Epigenetic Mechanisms That Influence Microvascular Complications

### Epigenetic Mechanisms in Diabetic Nephropathy

DN is a common microvascular complication of DM characterized by tubular interstitial fibrosis, glomerular mesangial hypertrophy and expansion, glomerular hyperfiltration with microalbuminuria and podocyte foot process effacement. The pathogenesis of DN is still not fully understood, but it has become increasingly clear that epigenetic mechanisms are involved in its development. Podocyte cells have been reported to play a crucial role in maintaining the structure and function of the glomerular filtration barrier, which is related to the pathogenesis of DN. Zhang et al. explored the effect of DNA methylation in murine diabetic podocytes and indicated that the expression of Dnmt1, nuclear factor Sp1 and NF-κB-p65 was markedly upregulated in podocytes under diabetic conditions, while inhibition of DNA methylation attenuated albuminuria, glomerular hypertrophy, mesangial matrix expansion and podocyte injury. Further mechanistic research found that increased Sp1 could bind to the *Dnmt1* promoter region and interact with NF-κB-p65 in the nucleus of HG-treated podocytes to participate in the regulation of Dnmt1. Thus, the Sp1/NF-κB-p65-Dnmt1 pathway may be a potential therapeutic target to protect against podocyte injury in DN (47). Recently, Hishikawa et al. found that lysine acetyltransferase 5 (KAT5)-mediated DNA damage repair associated with the DNA methylation status is essential for the maintenance of kidney podocytes. Podocyte-specific *Kat5*-knockout mice presented with severe albuminuria concomitant with increased DNA double-strand breaks and augmented DNA methylation in the nephrin promoter region (48). Aberrant DNA methylation, such as hypermethylation of kinesin family member 20B (*Kif20b*), claudin-18 (*Cldn18*), and solute carrier organic anion transporter family member 1a1 (*Slco1a1*), as well as hypomethylation of angiotensinogen (*Agt*), ATP-binding cassette sub-family C member 4 (*Abcc4*), cytochrome P450 4A10 (*Cyp4a10*), and *Glut5* were also observed in the proximal tubules of diabetic mice, which could lead to continuous mRNA expression of select genes to alter the phenotype of the proximal tubules in DN (49).

Histone modifications should also be kept in mind when considering to therapeutically target epigenetics to improve DN. DM is an inflammatory disease that releases inflammatory cytokines to promote the aggregation of leukocytes by innate cells in the diabetic kidney, which facilitates progressive fibrosis in advanced stages of DN. One study concentrated on the effect of glucose on the expression and histone modifications of a proinflammatory gene, thioredoxin-interacting protein (*TXNIP*) and found that glucose could induce the expression of *TXNIP* to enhance the development of DN through histone acetylation (50). Importantly, DM is also an endothelial disease. Endothelial activation refers to the endothelial expression of cell surface adhesion molecules that promote leukocyte recruitment. Alghamdi et al. have shown that the phosphorylation of histone H3 on serine residue 10 plays an important role in mediating endothelial activation in DN (51). TGF-β can alter pivotal chromatin histone modifications at target gene promoters to modulate gene expression in mesangial cells. TGF-β has been shown to inhibit H3K27me3 to promote pathological gene-mediated glomerular mesangial dysfunction and DN through the dysregulation of associated histone modifying enzymes and miRNAs (52). The 12/15-lipoxygenase (12/15-LO) is implicated in TGF-β-associated signaling, histone modifications and lysine methyltransferase Set7 regulation in the progression of DN. Knockout of the 12/15-LO (*Alox15*) gene inhibited TGF-β-induced expression of Set7 (*Setd7*) gene and pro-fibrotic genes, in kidneys of STZ-induced diabetic mice (53).

Recently, accumulating evidence has highlighted key roles of lncRNAs in the pathophysiology of DN. For example, lncRNA nuclear-enriched abundant transcript 1 (NEAT1) increases proliferation and fibrosis in glucose-induced mouse mesangial cell model by activating the protein kinase B/mTOR signaling pathway (54). With an opposite effect, overexpression of lncRNA CYP4B1-PS1-001 suppressed proliferation and fibrosis of mesangial cells under HG conditions (55). Moreover, lncRNAs are also confirmed to be involved in the regulation of podocyte injury. LncRNA LINC01619, as a competing endogenous RNA, modulated endoplasmic reticulum (ER) stress and podocyte injury in DN *via* the miR-27a/FOXO1 pathway (56). Another lncRNA, MALAT1, an important oncogene in numerous cancers, was increased in the kidney of STZ-induced diabetic mice and was dysregulated in HG-treated podocytes. However, knocking it down under HG conditions reversed podocyte damage *via* the downregulation of serine/arginine-rich splicing factor 1 overexpression, a MALAT1-binding protein, and partial inhibition of β-catenin nuclear accumulation (57).

### Epigenetic Mechanisms in Diabetic Retinopathy

DR is another severe microvascular complication in patients with DM, and is the leading cause of blindness. Numerous studies have focused on the roles of epigenetic modifications in DR and it has been reported that epigenetic modifications could serve as possible biomarkers for DR (58). An increase in oxidative stress has been considered a significant factor contributing to the development of DR. Ras-related C3 botulinum toxin substrate 1 (Rac1)-mediated cytosolic ROS production and the subsequent oxidative stress play key roles in mitochondrial injury and capillary cell apoptosis, which are associated with the DNA methylation status of the *Rac1* promoter (59). Moreover, a decrease in manganese superoxide dismutase (MnSOD), an antioxidant enzyme, has been observed in DR. Further investigation regarding the underlying epigenetic mechanisms discovered that hyperglycemia increased acetyl-H3K9, H4K20me3, and NF-kB-p65 at the promoter and enhancer regions of retinal *Sod2*, and upregulated protein and gene expression of *SUV420h2,* one of the primary enzymes for the trimethylation of H4K20. However, silencing *SUV420h2* by its siRNA in retinal endothelial cells blocked HG-induced increase in H4K20me3 at the *Sod2* enhancer and decrease in *Sod2* transcripts (60). Another study showed that hyperglycemia increased the binding of the histone demethylase lysine-specific demethylase-1 (LSD1) and Sp1 at *Sod2*, and decreased monomethyl H3K4 and dimethyl H3K4. Knocking down LSD1 with siRNA improved the HG-induced H3K4 demethylation at *Sod2* to upregulate *Sod2* gene expression (61). These findings indicate that epigenetic modifications play key roles in the regulation of retinal *Sod2* in the development of DR.

The activity and transcription of matrix metalloproteinase 9 (MMP-9) has also been observed to be increased in DR, which could damage retinal mitochondria and enhance oxidative stress. Additional epigenetic studies have demonstrated that glucose increased the binding of Dnmt1 and hydroxymethylase ten-eleven translocation 2 (Tet2) to the *MMP-9* promoter region in retinal endothelial cells. While Dnmt1 adds a methyl group to the cytosine forming methyl cytosine, Tet2 hydroxymethylates that cytosine to form 5-hydroxymethyl cytosine, in turn, activates *MMP-9* transcription. These changes were reversed with the MnSOD mimesis, MnTBAP, which regulated *MMP-9* transcription and improved mitochondrial damage (62, 63). The *MMP-9* promoter has also been reported to undergo histone modifications in DR. Hyperglycemia increased the levels of H3K27me3 and recruitment of enhancer of zeste homolog 2 (EZH2) at the *MMP-9* promoter. EZH2 suppression could reduce recruitment of Dnmt1 and Tet2 at the same promoter region of *MMP-9* to reduce its transcription and mitochondrial damage (64). Additionally, mitochondrial DNA (mtDNA) is injured with elevated base mismatches and hypermethylated cytosines (65). HG conditions reduced MutL homolog 1 (Mlh1) mitochondrial localization, an enzyme responsible for repairing the mismatched bases, and hypermethylated its promoter with increased Dnmt1 binding and decreased Sp1 binding. Inhibition of Dnmt1 could reduce hypermethylation of the *Mlh1* promoter, elevate its gene transcripts and decrease mtDNA mismatches, suggesting that the regulation of DNA methylation has a potential role to prevent mtDNA damage and slow or inhibit the development of DR (66).

Recent research has revealed regulatory roles of lncRNAs on inflammation in DR. The lncRNA MALAT1 was upregulated in the vitreous humors from diabetic patients and could impact the expression of inflammatory cytokines *via* its correlation with components of the polycomb repressive complex 2. Moreover, increased MALAT1 and related inflammatory transcripts in human retinal endothelial cells (HRECs) were detected following inhibition of Dnmts. However, HG treatment could not induce significant methylation changes in CpG sites across the *MALAT1* gene (67). Therefore, the mechanism of HG-induced overexpression of MALAT1 still remains to be investigated. In addition, HG conditions facilitated cell apoptosis and attenuated the cell activity concomitant with enhanced binding activity between NF-κB and the lncRNA MIAT. Further investigation revealed that MIAT could regulate miR-29b and subsequently regulate cell apoptosis in HG-treated rat retinal Müller cells (68). Due to the key role of epigenetics in diabetic microvascular complications, such as DN and DR, it is essential to explore how changes to the epigenome influence the etiology and pathogenesis of diabetic microvascular complications for the development of novel biomarkers and drug targets.

## The Role of SIRT1 in Microvascular Complications

### SIRT1 in Diabetic Nephropathy

Hasegawa et al. found that SIRT1 levels in proximal tubules were decreased prior to the development of albuminuria in STZ-induced diabetic mice. Moreover, knockout of *Sirt1* specifically in the proximal tubules worsened the DM-induced glomerular changes. Importantly, nondiabetic proximal tubule-specific *Sirt1* knockout mice developed albuminuria, suggesting that Sirt1 expression in the proximal tubules plays a necessary role for glomerular function. The expression of the tight junction protein CLDN1 has been shown to activate β-catenin-Snail pathway to induce podocyte effacement and cause albuminuria. However, overexpression of SIRT1 was able to blunt HG-induced upregulation of CLDN1 *via* the deacetylation of histones H3 and H4 with subsequent CpG methylation of *Cldn1* by recruiting Dnmt1 in human-derived renal epithelial cells (69). p66Shc, a biomarker for renal oxidative injury, is upregulated in DN. SIRT1 activation can inhibit p66Shc expression to improve DN-induced oxidative injury by facilitating the binding of SIRT1 to the *p66Shc* promoter and deacetylation of acetyl-H3 in human proximal tubular epithelial cell line (HK-2) under HG conditions (70). A recent study revealed that the repression of *SIRT1* transcription by HG conditions in renal tubular epithelial cells was dependent on the epigenetic regulation of hypermethylated in cancer 1 (HIC1), which could increase the levels of ROS and contribute to the development of DN. Mechanistically, HIC1 repressed *SIRT1* transcription in response to HG stimulation through an interaction with EZH2, an H3K27 trimethyltransferase, as well as Dnmt1 (71).

Moreover, interactions between SIRT1 and miRNAs have been reported to play an important role during DN therapy. One study that focused on the role of SIRT1 in HG-induced renal tubular epithelial injury showed that overexpression of SIRT1 decreased activity of NF-κB to upregulate miR-29 expression. NF-κB was demonstrated to downregulate miR-29 expression by directly binding to its promotor. Overexpression of miR-29 directly targeted Kelch-like ECH-associated protein 1 (Keap1) mRNA to decrease Keap1 expression and subsequently increase the expression of nuclear factor erythroid 2-related factor 2 (Nrf2) and downstream antioxidases, including glutathione S-transferase (GST) and nicotinamide adenine dinucleotide phosphate (NADPH) quinone dehydrogenase 1 (NQO1), which improved HG-induced renal tubular epithelial injury (72). In addition, HG conditions can elevate the levels of miR-34a-5p to exacerbate fibrosis by targeting SIRT1 in HK-2 cells (73). Overexpression of long intergenic ncRNA (lincRNA) 1700020I14Rik has been verified to interact with miR-34a-5p through direct targeting as well as an argonaute-2 dependent manner to inhibit cell proliferation and fibrosis through the SIRT1/hypoxia-inducible factor-1α (HIF-1α) signaling pathway during the progression of DN (74). Recently, Ge et al. found that lncRNA growth arrest special 5 (GAS5) could also upregulate SIRT1 expression to inhibit cell proliferation and fibrosis in DN by acting as an miR-221 sponge (75). Furthermore, inhibition of miR-133b and miR-199b upregulated SIRT1, which in turn attenuated TGF-β1-induced endothelial to mesenchymal transition and renal fibrosis in DN (76). Another lncRNA, SOX2-overlapping transcript (SOX2OT), was significantly downregulated in HG-treated human podocyte cells (HPCs). The overexpression of SOX2OT markedly improved the HG-induced HPC injury and increased the expression of Beclin-1 and the microtubule-associated proteins 1A/1B light chain 3-II (LC3-II) to LC3-I ratio, whereas decreased the levels of p62 by sponging miR-9 to facilitate SIRT1 expression (77). miR-155-5p also has been reported to regulate autophagy, which might be upregulated in patients with DN. Under HG conditions, inhibition of miR-155-5p could stimulate SIRT1 expression to promote autophagy by reducing binding to the SIRT1 3’ untranslated region in HK-2 cells (78).

In addition, SIRT1 can regulate oxidative stress, inflammation, apoptosis and fibrosis to improve DN by mediating the expression of several downstream targets. Excessive mitochondrial ROS production is considered an initiating event in the development of DN. Zhang et al. demonstrated that SIRT1 activation by resveratrol could inhibit mitochondrial ROS production, improve respiratory chain complex I and III activity and elevate the mitochondrial membrane potential in podocytes exposed to HG conditions, which was related to upregulation of PGC-1α expression (79). Consistent with this study, salidroside (broad spectrum bioactive effects) improved DN-induced renal structure damage probably by stimulating SIRT1/PGC-1α-mediated mitochondrial biogenesis (80). NADPH oxidase 4 (NOX4), a main enzyme contributing to increased oxidative stress, is upregulated in HG conditions. Treatment with puerarin (naturally occurring isoflavonoid) suppressed NOX4 expression to exert its anti-oxidative effect in HG-treated podocytes, which was dependent on SIRT1 expression (81).

**Figure 2:**
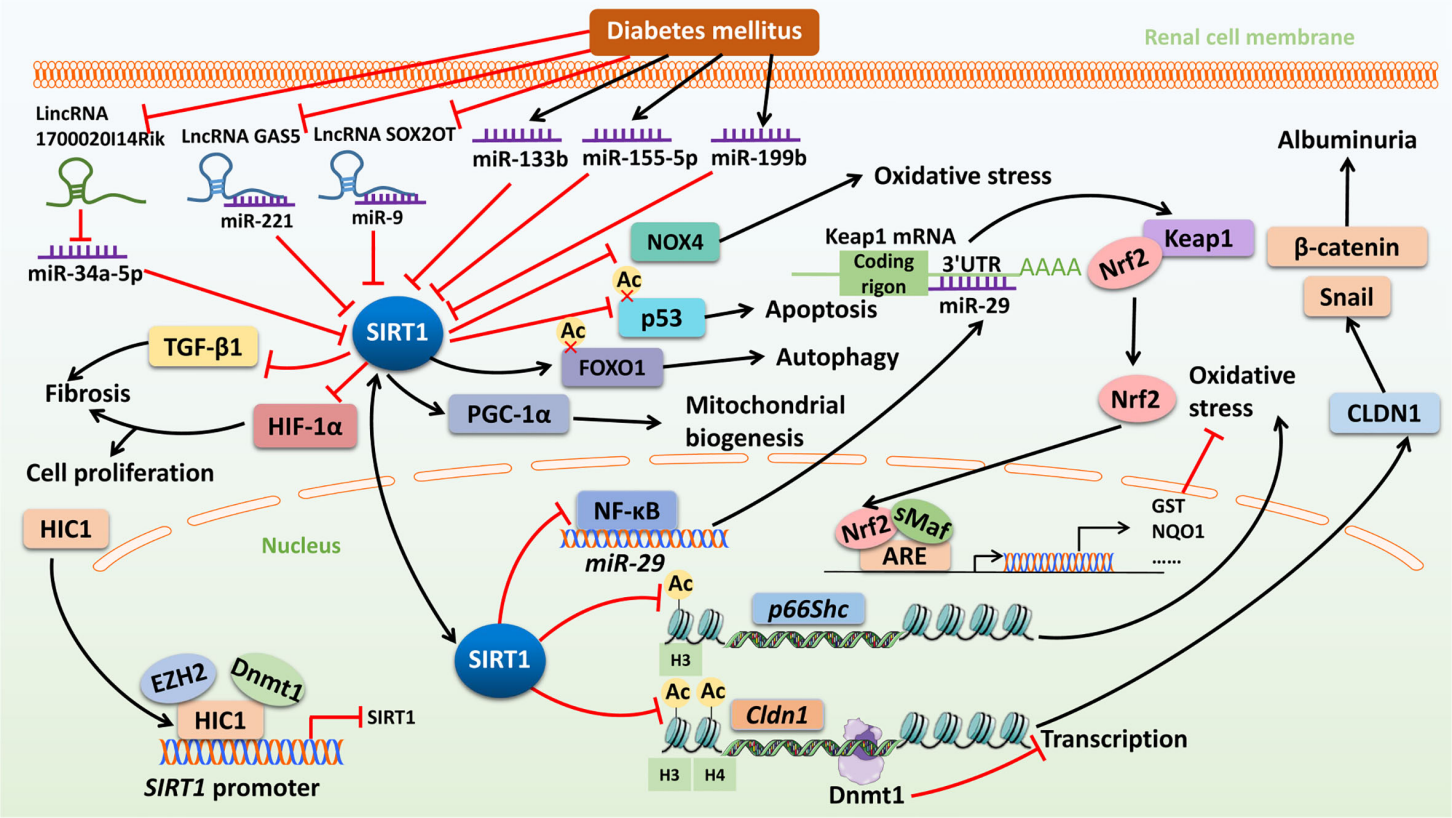
The mechanisms of action and signaling pathways of SIRT1 in diabetic nephropathy. Diabetes mellitus (DM) can inhibit SIRT1 expression by increased expression of miR-34a-5p, miR-221, miR-9, miR-133b, miR-155-5p, and miR-199b. HIC1 can repress *SIRT1* transcription in response to HG stimulation *via* an interaction with EZH2 and Dnmt1. Overexpression of SIRT1 can blunt CLDN1/β-catenin-Snail-mediated albuminuria in DN *via* the deacetylation of histones H3 and H4 with subsequent CpG methylation of *Cldn1* by recruiting Dnmt1. SIRT1 activation can inhibit p66Shc expression to improve oxidative stress in DN by deacetylation of acetyl-H3. SIRT1 activation can upregulate miR-29 expression by reduction of NF-κB binding to its promotor. Overexpression of miR-29 directly targets Keap1 mRNA to decrease Keap1 expression and subsequently increase Nrf2 expression. Free Nrf2 translocates to the nucleus, where it dimerizes with members of the sMaf family and binds to ARE within regulatory regions of a wide variety of cell defense genes, including *GST* and *NQO1*. LincRNA 1700020I14Rik can interact with miR-34a-5p to inhibit cell proliferation and fibrosis in DN though the SIRT1/HIF-1α signaling pathway. LncRNA GAS5 and SOX2OT can upregulate SIRT1 expression by sponging miR-221 and miR-9, respectively. SIRT1 can inhibit TGF-β1-induced renal fibrosis and NOX4-induced oxidative stress, and stimulate PGC-1α-mediated mitochondrial biogenesis to improve DN. Finally, SIRT1 can deacetylate and activate FOXO1 to upregulate the level of autophagy, and deacetylate and inhibit p53 to inhibit apoptosis, thus improving DN. SIRT1, Sirtuin 1; DM, diabetes mellitus; miR, microRNA; HIC1, hypermethylated in cancer 1; HG, high glucose; EZH2, enhancer of zeste homolog 2; Dnmt1, DNA methyltransferase 1; CLDN1, claudin-1; DN, diabetic nephropathy; NF-κB, nuclear factor kappa B; Keap1, Kelch-like ECH-associated protein 1; Nrf2, nuclear factor erythroid 2-related factor 2; sMaf, small Maf; ARE, antioxidant response element; GST, glutathione S-transferase; NQO1, nicotinamide adenine dinucleotide phosphate (NADPH) quinone dehydrogenase 1; LincRNA, long intergenic non-coding RNA; HIF-1α, hypoxia-inducible factor-1α; LncRNA GAS5, long non-coding RNA growth arrest special 5; SOX2OT, SOX2-overlapping transcript; TGF-β1, transforming growth factor-beta 1; NOX4, NADPH oxidase 4; PGC-1α, peroxisome proliferator-activated receptor gamma coactivator-1 alpha FOXO1, forkhead box O 1; p53, tumor protein 53.

Furthermore, inhibition of inflammation is one of the important mechanisms for SIRT1 to protect the kidney from injury. Du et al. found that Tangshen formula (traditional Chinese herbal medicine for treatment of kidney disease) activated SIRT1 to reduce the expression of the proinflammatory factors NF-κB and monocyte chemoattractant protein-1, which improved the severity of DN (82). NF-κB also has been shown to regulate autophagy, which plays a crucial role in several kidney diseases including DN. Hyperglycemia downregulates the levels of Beclin 1 and LC3-II to induce renal dysfunction, which can be reversed by SIRT1-mediated deacetylation of NF-κB-p65 (83). The SIRT1-FOXO1 autophagy signal axis also plays a key role in the regulation of autophagy in DN. Xu et al. reported that metformin (used to treat T2D) upregulated the levels of autophagy to alleviate oxidative stress in renal tissue, and reduced pathological and structural changes of glomeruli through the deacetylation and activation of FOXO1 by SIRT1 (84). Furthermore, SIRT1 plays an important anti-apoptotic role in the treatment of DN. Wang et al. reported that SIRT1 activation by resveratrol could deacetylate p53 to improve the renal tubular injury induced by hyperglycemia through the inhibition of apoptosis (9). The summarizing schematic diagram about the diverse mechanisms and pathways of SIRT1 in DN is presented in **Figure 2**.

### SIRT1 in Diabetic Retinopathy

Diabetic wild-type mice present with increased cell apoptosis, degenerative capillaries, and decreased vascular density accompanied with hypermethylation at the promoter of *Sirt1* in retinal microvessels, but overexpression of SIRT1 ameliorated these pathological changes in DR. Overexpression of SIRT1 also protected mitochondria from DM-induced mtDNA damage and prevented the activation of mitochondria-damaging MMP-9. Mechanistically, DM suppresses *Sirt1* transcription by regulating the DNA methylation of its promoter *via* increasing Dnmt1 expression. Importantly, Dnmt1 expression was downregulated in diabetic *Sirt1* mice through decreased H3K9 acetylation of the *Dnmt1* promoter, suggesting an important role for epigenetics in the transcription of *Sirt1* (85). Furthermore, upregulation of Sirt1 by resveratrol led to increased deacetylation of NF-κB-p65 that reduced binding of NF-κB-p65 at the *MMP-9* promoter to prevent mitochondrial damage and the development of DR (86).

ncRNAs can serve as biomarkers for various pathological conditions and participate in the initiation and progression of DR by targeting SIRT1. Zhao et al. investigated the important effect of miR-23b-3p on metabolic memory in DR. They found that the expression of miR-23b-3p was promoted by HG treatment and remained elevated even after the return to normal levels of glucose, whereas the expression of *SIRT1* was decreased in HRECs. Further investigation indicated that NF-κB-p65 could mediate HG-induced transactivation of miR-23b-3p by binding to the promoter element of *pri-miR-23b-27b-24-1*. However, suppression of miR-23b-3p expression could inhibit acetyl-NF-κB expression, which was abolished by *SIRT1* knockdown in HG-treated HRECs. These findings suggest that the miR-23b-3p/SIRT1/NF-κB feedback loop may exert a crucial role in the establishment and maintenance of metabolic memory in DR (87). In addition, inhibition of miR-211, miR-29b-3p, miR-221, miR-34a, miR-217, miR-195, miR-377, miR-543, and miR-204 was also reported to upregulate the expression of SIRT1 and improve DR by alleviating apoptosis, inflammation, oxidative stress, angiogenesis, and ER stress (88–97).

LncRNAs can also regulate the expression of miRNAs to participate in the progression of DR. For instance, lncRNA maternally expressed gene 3 (MEG3) alleviated HG-induced inflammation and apoptosis in retina epithelial cells *via* the inhibition of NF-κB signaling and the increase in Bcl-2/Bcl-2-associated X protein ratio by regulating the miR-34a/SIRT1 axis (91). Recently, Ke et al. reported that lncRNA small nucleolar RNA host gene 7 (SNHG7) could inhibit miR-543 to upregulate SIRT1 expression, thereby inhibiting HG-induced angiogenesis in HRECs (95). The role of ncRNAs-mediated SIRT1 changes in DR are summarized in **Table 1**.

**Table 1:** The role of ncRNA-mediated SIRT1 changes in diabetic retinopathy.

ncRNAs	Model	Effect on expression of SIRT1	Functions regulated	References
miR-211	STZ-induced diabetic Wistar rats and HUVEC	Inhibition	Apoptosis	Liu et al. (88)
miR-29b-3p	HRMEC	Inhibition	Apoptosis	Zeng et al. (89)
miR-221	HRMEC	Inhibition	Apoptosis	Chen et al. (90)
miR-34a	ARPE-19	Inhibition	Inflammation and apoptosis	Tong et al. (91)
miR-217	ARPE-19	Inhibition	Inflammation and apoptosis	Xiao et al. (92)
miR-195	STZ-induced diabetic Sprague-Dawley rats, HRMEC and HMEC	Inhibition	Oxidative stress and production of ECM proteins	Mortuza et al. (93)
miR-377	HREC	Inhibition	Angiogenesis and inflammation	Cui et al. (94)
miR-543	HREC	Inhibition	Angiogenesis	Ke et al. (95)
miR-204	PRPEC and ARPE-19	Inhibition	ER stress and apoptosis	Peng et al. (96)
miR-204	High-fat diet and STZ-induced mice and MIO-M1	Inhibition	Inflammation	Tu et al. (97)
LncRNA MEG3	ARPE-19	Activation	Inflammation and apoptosis	Tong et al. (91)
LncRNA MEG3	High-fat diet and STZ-induced mice and MIO-M1	Activation	Inflammation	Tu et al. (97)
LncRNA SNHG7	HREC	Activation	Angiogenesis	Ke et al. (95)

SIRT1, Sirtuin 1; ncRNA, non-coding RNA; miR, microRNA; LncRNA MEG3, long non-coding RNA maternally expressed gene 3; SNHG7, small nucleolar RNA host gene 7; ECM, extracellular matrix; ER, endoplasmic reticulum; STZ, streptozocin; EC, epithelial cell; HUVEC, human umbilical vein EC; HRMEC, human retinal microvascular EC; ARPE-19, human retinal pigment EC; HREC, human retinal EC; HMEC, human dermal microvascular EC; PRPEC, primary retinal pigment EC; MIO-M1, human Müller cell line.

These above-mentioned findings highlight the importance for us to continue to study the roles of SIRT1 in diabetic microvascular complications. The related epigenetic mechanisms of SIRT1 may further provide new insights into potential therapeutic strategies for the treatment of diabetic microvascular complications.

## SIRT1 as a Potential Drug Target for the Treatment of Diabetic Complications

Given that SIRT1 is a key mediator to prevent the progression of diabetic complications, it is necessary to develop specific therapeutic strategies to restore SIRT1 activity. The most obvious approach would be to simply stimulate SIRT1 activity using SIRT1 agonists. Resveratrol is a well-known polyphenolic SIRT1 agonist that has been reported to ameliorate diabetic complications through the regulation of autophagy, oxidative stress and mitochondrial function in DCM, DN and DR animal models (9–12, 79, 86). Milne et al. have shown small molecule activators of SIRT1, including SRT1460, SRT1720, and SRT2183, are structurally unrelated to resveratrol but can be 1,000-fold more potent as activators compared to resveratrol. These compounds can bind to the SIRT1 enzyme-peptide substrate complex at an allosteric site in the amino-terminal region to the catalytic domain and lower the Michaelis constant for acetylated substrates. Additional investigation found that these compounds ablated insulin resistance in mice with diet-induced obesity and genetically obese mice (Lep^ob/ob^), and improved insulin sensitivity and glucose homeostasis in key metabolic tissues including liver, muscle, and fat of male fatty Zucker rats (98). However, recent studies have indicated that resveratrol, SRT1460, SRT1720, and SRT2183 may not be direct activators of SIRT1 (99, 100).

Another SIRT1 agonist, N-acetyl-5-methoxytryptamine, commonly known as melatonin, has been revealed to exert its protective properties in DCM through the prevention of Drp1-mediated mitochondrial fission by SIRT1/PGC1-α pathway (37). Another study showed that melatonin could also inhibit pro-inflammatory cytokine production in the progression of DR (97). Recently, Xue et al. showed that salidroside, an active component of the Traditional Chinese Medicinal plant *Rhodiola rosea* L., also activated SIRT1/PGC1-α signaling pathway to stimulate mitochondrial biogenesis, thus improving DN (80). Other herbal medicines or compounds, such as puerarin, Tangshen formula, astragaloside IV, and astragalus polysaccharide, have also been reported to ameliorate diabetic complications *via* the activation of SIRT1 (81–83, 96). Metformin, a derivative of biguanides, is a first line drug for the therapy of T2D and it has been reported to alleviate DN by inducing the SIRT1/FOXO1 autophagic signaling axis (84). Moreover, a study focused on the protective properties of acetaldehyde dehydrogenase 2 (ALDH2), a rate-limiting enzyme for alcohol metabolism, and found that ALDH2 could improve damage induced by STZ in rats with aged diabetic retinas (101). Recently, Qu et al. indicated that 1,25(OH)_2_D_3_, as a potential PARP1 inhibitor, played a crucial protective role in DCM through PARP1/SIRT1/mTOR-related mechanisms (40).

In summary, SIRT1 agonists are promising candidates to use in a novel therapeutic approach for the treatment of diabetic complications. Although the effects of numerous SIRT1 agonists on diabetic complications have been reported, the specificity of the SIRT1 agonists remains a concern. Further research is required to investigate specific SIRT1 agonists and push them closer to clinical application. SIRT1 agonists and their related roles in diabetic complications are summarized in **Table 2**.

**Table 2:** SIRT1 agonists and their respective roles in diabetic complications.

Approaches	Diseases	Mechanisms regulated	Functions regulated	References
Resveratrol	DCM	SIRT1/FOXO1/Rab7	Autophagy	Wang et al. (10)
	DCM	Deacetylation of NF-κB-p65 and histone H3	Oxidative stress	Bagul et al. (11)
	DCM	SIRT1/PGC‐1α	Mitochondrial function	Fang et al. (12)
	DN	SIRT1/PGC‐1α	Mitochondrial oxidative stress	Zhang et al. (79)
	DN	SIRT1/p53	Apoptosis	Wang et al. (9)
	DR	SIRT1/MMP-9	Mitochondria damage	Kowluru et al. (86)
SRT1460	Diet-induced obesity mice, *Lep^ob/ob^* mice and male fatty Zucker rats	SIRT1	Insulin sensitivity and glucose homeostasis	Milne et al. (98)
SRT1720	Diet-induced obesity mice, *Lep^ob/ob^* mice and male fatty Zucker rats	SIRT1	Insulin sensitivity and glucose homeostasis	Milne et al. (98)
SRT2183	Diet-induced obesity mice, *Lep^ob/ob^* mice and male fatty Zucker rats	SIRT1	Insulin sensitivity and glucose homeostasis	Milne et al. (98)
Melatonin	DCM	SIRT1/PGC1-α	Drp1-mediated mitochondrial fission	Ding et al. (37)
	DR	LncRNA MEG3/miR‐204/SIRT1	Inflammation	Tu et al. (97)
Salidroside	DN	SIRT1/PGC1-α	Mitochondrial biogenesis	Xue et al. (80)
Puerarin	DN	SIRT1/NOX4	Oxidative stress	Li et al. (81)
Tangshen formula	DN	SIRT1/NF−κB	Inflammation	Du et al. (82)
Astragaloside IV	DN	SIRT1/NF−κB-p65	Autophagy	Wang et al. (83)
Astragalus polysaccharide	DR	miR-204/SIRT1	ER stress and apoptosis	Peng et al. (96)
Metformin	DN	SIRT1/FOXO1	Autophagy	Xu et al. (84)
ALDH2	DR	SIRT1/Nrf2	Oxidative stress	He et al. (101)
1,25(OH)_2_D_3_	DCM	PARP1/SIRT1/mTOR	Cardiac dysfunction, hypertrophy and fibrosis	Qu et al. (40)

SIRT1, Sirtuin 1; ALDH2, acetaldehyde dehydrogenase 2; 1,25(OH)_2_D_3_, 1,25-Dihydroxyvitamin D3; DCM, diabetic cardiomyopathy; DN, diabetic nephropathy; DR, diabetic retinopathy; Lep^ob/ob^, genetically obese mice; FOXO1, forkhead box O 1; Rab7, Ras-related protein Rab-7a; NF-κB, nuclear factor kappa B; PGC-1α, peroxisome proliferator-activated receptor-gamma coactivator-1 alpha; p53, tumor protein 53; MMP-9, matrix metalloproteinase 9; LncRNA MEG3, long non-coding RNA maternally expressed gene 3; miR, microRNA; NOX4, nicotinamide adenine dinucleotide phosphate oxidase 4; Nrf2, nuclear factor erythroid 2-related factor 2; PARP1, poly (ADP-ribose) polymerase 1; mTOR, mammalian target of rapamycin; Drp1, dynamin-related protein 1; ER, endoplasmic reticulum.

## Conclusion

Advances in the field of epigenetics have deepened and aided our understanding of gene regulation in health and disease. The evidence discussed above suggests that epigenetic mechanisms play crucial roles in the pathophysiological process of diabetic complications. Further study of epigenetic regulatory events will help us predict the onset or progression of diabetic complications more accurately. Therefore, therapeutic options based on epigenetic regulation may provide a unique treatment strategy whereby physiological gene expression patterns can be recovered and the progression of diabetic complications can be prevented. SIRT1 as a deacetylase has been found to exert protective roles in diabetic complications. To date, research regarding the important role of SIRT1 in reducing diabetic complications is underway but the exact effects in clinical applications have not been fully established due to the intricacy of the regulatory mechanisms and the multiple pathways involved. Therefore, the related epigenetic regulation of SIRT1 for the clinical treatment of diabetic complications is still a long way off. Additional research is required to identify the causal relationship between epigenetic mechanisms and disease status, and the precise mechanisms of SIRT1 involvement in epigenetic regulation for the treatment of diabetic complications require further investigation.

## Author Contributions

JW(a), JW(b), MX, and YG performed the systematic search, did data extraction, interpreted the data and drafted the review. SW, YT, and JZ contributed to the discussion. JG supervised and revised the manuscript. All authors contributed to the article and approved the submitted version.

## Funding

This study was supported by the Qilu Young Scholar’s Program of Shandong University (21330089963007), the National Natural Science Foundation of China (81700329, 81770375), and the Jilin Science and Technology Department (20200801061GH).

## Conflict of Interest

The authors declare that the research was conducted in the absence of any commercial or financial relationships that could be construed as a potential conflict of interest.

The handling editor declared a shared affiliation with several of the authors JW(a), JW(b), MX, YG, YT, and JG at time of review.
